# Effect of active learning and online discussions on the academic performances of dental students

**DOI:** 10.1186/s12909-022-03377-9

**Published:** 2022-04-25

**Authors:** Jaeseo Lim, Hyunwoong Ko, Jooyong Park, Jungjoon Ihm

**Affiliations:** 1 Interdisciplinary Program in Cognitive Science, Seoul National University, Seoul, 08826 Republic of Korea; 2 Department of Psychiatry, Seoul National University College of Medicine & SMG-SNU Boramae Medical Center, Seoul, Republic of Korea; 3 Department of Psychology, Seoul National University, Seoul, 08826 Republic of Korea; 4 Dental Research Institute, Seoul National University School of Dentistry, Seoul, 03080 Republic of Korea

**Keywords:** Discussion, Active learning, Online learning, Self-study, Health professions education

## Abstract

**Background:**

COVID-19 caused significant confusion around the world, and dental education was no exception. Therefore, in line with the demands of the times, this study sought to determine the applicability of online active learning to dental education.

**Methods:**

This study was conducted in the second semester of 2020 at a school of dentistry in a selective university in Korea. A total of 114 dental students were recruited. Participants were assigned to four different groups (lecture and discussion [LD], lecture and discussion with instructor’s worksheet [LW], self-study and discussion [SSD], and self-study and discussion with instructor’s worksheet [SW]) using the random breakout room function in the Zoom video conference application. Their final test scores were then analyzed using analysis of variance and the online active learning results were compared with the offline learning results.

**Results:**

The scores were highest for the transfer type items in the SSD group, followed by the SW group and the two lecture groups, which had no significant differences. These scores and pattern differences between the groups were similar for all items. The results suggested that studying by oneself rather than simply listening to lectures enhanced the effects of the discussions and led to higher learning outcomes. In addition, the effect of the instructor's intervention in the middle of the discussion varied depending on the pre-learning activities of discussion. As with previous offline experiments, self-study followed by group discussion had higher learning outcomes for both the verbatim and transfer type items.

**Conclusions:**

In agreement with the Interactive, Constructive, Active, and Passive (ICAP) framework and other active learning theories, the findings clearly indicated that online active learning was applicable to dental students, and when self-study precedes discussion, the learning is richer and the learning outcomes are better.

## Background

The pandemic has meant that many countries have been forced to move to online education, which is learning that takes place regardless of spatial or temporal restrictions using digital tools such as the internet, smartphones, and other mobile devices [1]. While online learning definitions differ slightly depending on the context, there are several main types as follows: web-based learning, e-learning, cyberlearning, distance learning, and mobile learning [2, 3]. Some educational institutions also upload their lectures online for students to access through Google Classrooms, WebQuest, or other online learning platforms [4, 5]. In response to the COVID-19 pandemic, entire health education curricula, including subjects, such as basic science and behavioral science, were transitioned to online formats [6, 7].

Educators in the health professions have transformed pedagogy by reducing or even eliminating lectures [7, 8] and by implementing team-based, active, and self-directed learning [9–12]. In the past few years, educational stakeholders, including those in dental education, have been converting their classroom-style one-way lecture learning to asynchronous learning instruction [13–16]. Similarly, student-centered learning methods, such as discussion, rather than lectures are being implemented, and the learning effects has been confirmed in several comparative studies on the effectiveness of learning from one-way lectures or discussion-style videos [17, 18]; those who watched while discussing the content were found to have better performances. However, many students majoring in medicine and dentistry are still learning based on monologue-style lecture sessions, laboratory sessions, and simulations [7].

Previous research has explored the Interactive, Constructive, Active, and Passive (ICAP) framework, which describes four cognitive active learning engagement modes, and examined whether this framework could effectively train health professionals [8]. The passive mode generally refers to calmly sitting in traditional lectures, and the other three modes include segmented active learning methods. As the ICAP framework involves both interactive and active learning, prior studies that have explored the effectiveness of discussions on student learning within health-related subjects, such as medicine, dentistry, veterinary medicine, and nursing, confirmed that discussions were the most effective [8, 19, 20].

Past studies have been limited as the experiments were conducted only on unstructured discussions. [8] However, it has been found that teacher-facilitated, small group discussions can better promote high-level comprehension [21–24]. For example, Murphy et al. [21, 22] conducted a teacher-centered small group discussion approach called 'Quality Talk', in which the language and arts teachers provided short lectures on questions or arguments and then conducted text-based discussions in class. It was found from the video recording and discussion analyses that the students gradually increased their critical analytical thinking abilities and increased their exploratory conversation participation in response to the teachers’ questions. Langer also conducted an English achievement experiment with 44 teachers from 25 schools and 88 classrooms [23, 24]. She found that the teachers’ questions were very important in the discussions; when the teachers actively participated in the discussions, the students were better able to develop a multi-perspective understanding, which proved that the teachers’ participation in the discussions could increase the student learning achievements.

However, research into the feasibility of online discussions has not been conducted in health education contexts, with most previous online discussion research having focused on more qualitative domains [25–27], such as the increase in the students’ mutual communication skills [28], the increase in student participation rates, and the development of cooperative thinking skills [29]. Furthermore, most of the aforementioned studies were conducted offline, with few having focused on the relationship between online discussions and student learning achievements. Therefore, systematic online health education environment research is needed to assess whether such education is qualitatively comparable to offline education.

Therefore, the present study had the following research aims: (1) to determine whether the dental student learning outcomes from discussions were affected by preliminary learning activities; (2) to investigate the effect of instructor intervention on discussions based on the preliminary learning activities; and (3) to systematically examine and compare offline discussion data and the online discussion experiments to determine whether the existing active learning paradigm is also applicable to online learning.

An online study was conducted that provided active learning for dental students through the online video conferencing system Zoom. Based on the desirable active learning proposed in Chi and Wylie’s ICAP framework, learning activities with the same content before the discussions were compared. Because of the many findings in previous research that compared the learning from lectures and that from self-reading, [30, 31] it was predicted that the self-study mode would be more effective for learning than lecturing because it is more active and productive [19, 20].Whether the instructor’s intervention was effective when the learning activities (i.e., lecturing vs. self-studying) preceded the discussion was also assessed. Finally, an offline discussion experiment that had been conducted under similar conditions was systematically compared with the online discussion experiment in this study.

## Materials and methods

A predetermined sample size of at least 19 participants per group was decided on to ensure a reliable comparison could be made with the similar pre-COVID offline experiments [8] and a power analysis using G*Power was conducted to determine whether the experimental design had enough power [32]; we found that 19 participants provided 80% power to detect an effect size of 0.87 (alpha = 0.05).

### Participants and ethics

The participants (*N* = 114) were recruited from the school of dentistry at Seoul National University in Korea and the study was reviewed and approved by the Institutional Review Board of the School of Dentistry (approval No. S-D20200053). The undergraduate participants in the School of Dentistry were randomly divided into the following four online groups: lecture and discussion (LD); lecture and discussion with an instructor’s worksheet (LW); self-study and discussion (SSD); and self-study and discussion with an instructor’s worksheet (SW), and two offline groups: lecture and discussion (LD) and self-study and discussion (SSD). No significant difference was noted in age and gender between randomly assigned students to the group.

### Survey on prior knowledge

A survey was conducted to assess the participants’ prior knowledge or interest in the stimulus topic to minimize the background knowledge effect on the experiment [33, 34]. The six survey items were assessed using a 7-point Likert scale ranging from “have no idea (1)” to “know very well (7).” The survey on prior knowledge comprised six items. Two items were related to the learning content, and another four items were unrelated (i.e., the genome project, civil law, the legalization of same-sex marriage, and the Special Act on Sexual Violence).

### Lecture video with learning material and final test items

The learning materials used were the same as those for Lim et al., as acknowledged by the authors [8]. The 18-min lecture video used in this study was a monologue-style lecture on the topic of law, and particularly on the criminal procedure code, accusations, and charges. According to review by Barnett and Ceci [35] and Cook et al. [36] that a non-health profession topic can be used to measure the achievement of health profession students, we used an unrelated topic that was not covered in dental school. Thus, this topic was chosen because it was not typically taught in undergraduate courses, was a topic that the dental students were not familiar with, and to evaluate the student learning, the assessment included a finite set of verbatim answers as well as transfer type items. The seven-page learning materials contained content on the same topic along with the lecture video.

The final test questions included both verbatim and transfer type items [8]. The verbatim type items comprised 10 questions with either short or multiple-choice answers that could be directly inferred from the learning materials, each of which were worth 1 point, that is, a perfect score was 10. The transfer type items consisted of four questions, each requiring a total comprehension of the given materials and the application of these materials to new situations. The total value of the transfer type items was given as 15 points.

### Procedure

First, the participants completed a consent form and the survey on prior knowledge. Then, the participants were randomly assigned to four groups using the random breakout room function in Zoom. The LD group watched the 18-min lecture video and then discussed the content for 15 min in groups of three or four. The LW group also watched the 18-min lecture video; however, the instructors intervened using a worksheet in the online discussion. The SSD group participants studied the written material by themselves for 18 min and then discussed it for 15 min in groups of three or four. The SW group also studied the written material by themselves for 18 min, but the instructor intervened using the additional learning materials in the discussion section. The participants in the two self-study groups were allowed to take notes and underline the learning materials while they studied.

At the beginning of the discussion, the participants were told they had 15 min for the discussion, after which there would be a test. The participants were not allowed to ask questions when being given these instructions, that is, the researcher did not give any instructions and did not intervene during the discussion. After 15 min, all four groups took a 20-min test on the given material.

### Data analysis

The data analysis was conducted with SPSS version 23 (SPSS Inc., Chicago, IL, USA), which involved descriptive statistics, calculations for the means (Ms), standard deviations (SDs), minimums, and maximums. We conducted a two-way analysis of variance (ANOVA) by using a pre-discussion self-study, teacher intervention in the middle of discussion, and the interaction between the self-study and intervention as independent variables and students' learning outcomes as dependent variables, with a *P*-value < 0.05 considered to indicate statistically significant differences.

## Results

### Online active learning outcomes

The topics were law-related and dealt with accusations, charges, and the criminal procedure code. First, participants completed the survey on prior knowledge, and the six survey items were analyzed by topic as follows: criminal procedure code *(M* = 1.98, *SD* = 1.06), accusation and charge (*M* = 2.45, *SD* = 1.07), genome project (*M* = 3.73, *SD* = 1.93), civil law (*M* = 1.86, *SD* = 0.99), legalization of same-sex marriage (*M* = 2.47, *SD* = 1.06), and the Special Act on Sexual Violence (*M* = 2.64, S*D* = 1.47). The scores for the topic-relevant items (criminal procedure code, accusation, and charge) were significantly lower than for the genome project item; respectively, *t* (164) = 7.29, *P* < 0.001 and *t* (164) = 5.34, *P* < 0.001. However, no background knowledge differences were found between the four groups. Thus, the students had little background knowledge related to the learning topic, and little difference was noted in their prior knowledge level.

This analysis satisfied the assumptions of normality, homogeneity of variance, and independence, and then used ANOVA. The means and SDs for the total, verbatim type, and transfer type item scores are shown in Table 1. To reduce any scoring subjectivity, 15 final test papers (20% of the total) were randomly selected and marked by two raters. For all test items, the interrater agreement analysis measured using an intraclass correlation was 0.89 for the {ICC (3, *k*)}. As the agreement interrater agreement measured by the ICC was good, the remaining final test papers were marked by the first author.

**Table 1:** Learning outcome comparison for item types and study conditions

Type of item	LD (*n* = 21)	LW (*n* = 19)	SSD (*n* = 22)	SW (*n* = 20)	*P*
Total score (25 points)	10.19 (4.19)	9.89 (5.21)	16.55 (2.99)	12.30 (4.80)	< 0.001
Verbatim (10 points)	7.05 (2.67)	5.84 (2.64)	8.50 (1.22)	6.90 (1.92)	0.002
Transfer (15 points)	3.14 (2.74)	4.05 (3.31)	8.05 (2.62)	5.20 (2.61)	< 0.001

Data are shown as mean values (standard deviations)

*LD* Lecture and Discussion, *LW* Lecture and discussion with Worksheet, *SSD* Self-study and Discussion, and *SW* Self-study and discussion with Worksheet

For each group, total, verbatim type, and transfer type item scores are given

**Fig. 1:**
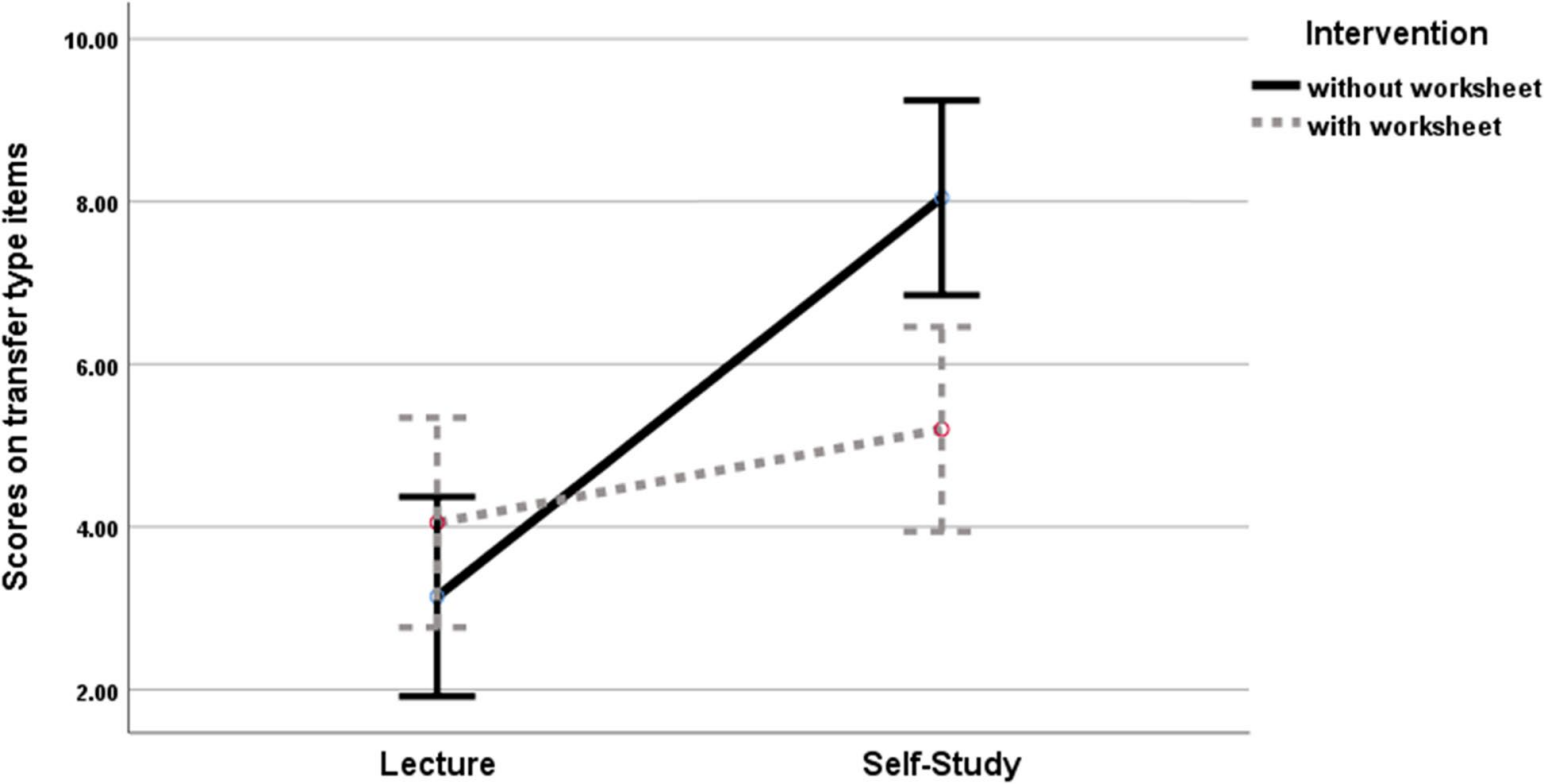
Main effects and interaction effect for the transfer type items. *Note*. Error bars represent 95% confidence interval

The main effect of study condition and the interaction effects between these two conditions (lecture versus. self-study) on the students' learning outcomes, and particularly on the transfer type items, were measured. The main effect of the study condition was found to be significant; *F* (1, 78) = 23.47, *P* < 0.001, partial eta-squared = 0.231. However, no significant difference was found for the main effect of the discussion condition (discussion with worksheet versus. free discussions); *F* (1, 78) = 2.40, *P* = 0.125, partial eta-squared = 0.030; however, the interaction effect of these two factors was significant; *F* (1, 78) = 9.04, *P* < 0.001, partial eta-squared = 0.104 (Fig. 1).

Significant differences were found for the total score (*F* (3, 78) = 12.35, *P* < 0.001, partial eta-squared = 0.322), verbatim type items (*F* (3, 78) = 5.22, *P* = 0.002, partial eta-squared = 0.167), and transfer type items (*F* (3, 78) = 12.21, *P* < 0.001, partial eta-squared = 0.320). Comparison analyses weres then conducted to determine if there were any differences between the groups based on the hypotheses. The SSD group had achieved significantly higher total scores than the SW, LD, and LW groups: *t* (78) = 3.42, *P* = 0.001, effect size Cohen’s d = 1.299; *t* (78) = 5.18, *P* < 0.001, *d* = 1.747; *t* (78) = 5.28, *P* < 0.001, *d* = 1.568, respectively. However, there were no significant differences between the LD and LW groups, between the LW and SW groups, or between the LD and SW groups; *t* (78) = 0.23, *P* = 0.817, *d* = 0.063; *t* (78) = 1.87, *P* = 0.066, *d* = 0.542; *t* (78) = 1.68, *P* = 0.097, *d* = 0.545, respectively.

The SSD group had significantly higher scores for the verbatim type items than the SW, LD, and LW groups: respectively, *t* (78) = 2.37, *P* = 0.020, *d* = 0.995; *t* (78) = 2.19, *P* = 0.032, *d* = 0.699; and *t* (78) = 3.90, *P* < 0.001, *d* = 1.298. However, there were no significant differences between the LD and LW groups, between the LW and SW groups, or between the LD and SW groups: respectively, *t* (78) = 1.75, *P* = 0.084, *d* = 0.457; *t* (78) = 1.52, *P* = 0.133, *d* = 0.460; and *t* (78) = 0.22, *P* = 0.829, *d* = 0.065.

The SSD group had significantly higher scores for the transfer type items than the SW, LD, and LW groups; respectively, *t* (78) = 3.26, *P* = 0.002, *d* = 1.088; *t* (78) = 5.69, *P* < 0.001, *d* = 1.828; and *t* (78) = 4.04,* P* < 0.001, *d* = 1.338. The SW group had significantly higher scores than the LD group, *t* (78) = 2.33, *P* = 0.022, *d* = 0.770; however, there were no significant differences between the LD and LW groups, or between the LW and SW groups; *t* (78) = 1.02, *P* = 0.312, *d* = 0.300; and *t* (78) = 1.27, *P* = 0.208, *d* = 0.386.

These results supported the our predictions that the discussion groups with preceding active learning activities (i.e., self-study) would have better learning outcomes, and the SSD group would have significantly higher scores than the LD group for the final test transfer items. Therefore, these empirical results were in line with the ICAP framework. Interestingly, it was found that the effects of the instructor’s intervention in the discussion were dependent on the pre-discussion learning activities. The LW group had higher scores than the LD group, and the SW group had lower scores than the SSD group, which indicated that when using discussions in the classroom, the instructors need to employ different types of intervention depending on the student learning environments or conditions.

### Comparison of offline active learning with online active learning

Additional analyses were systematically conducted to compare the dental students’ online and offline learning outcomes. To replicate the results of an offline experiment [8] and determine whether the same extent of active learning was possible using online discussion, the effects of the online discussion on the student’s learning outcomes, and especially the learning transfer, were examined. For this analysis, only the data from the dental students in Lim et al.’s experiment were compared with the discussion groups that had not had any interventions, that is, the LD and SSD groups.

The means and SDs for all groups are given in Table 2. The offline with online study results were compared between the groups that had had learning activities before the discussions, that was the lecture and self-study groups. Therefore, the four groups being compared were the lecture and discussion offline group (LD offline, *n* = 12), the lecture and discussion online group (LD online, *n* = 21), the self-study and discussion offline group (SSD offline, *n* = 20), and the self-study and discussion online group (SSD online, *n* = 22). A comparative analysis using ANOVA revealed that the groups had significant differences for the total scores, verbatim type items, and transfer type items; respectively, *F* (3, 71) = 21.13, *P* < 0.001, partial eta-squared = 0.472; and *F* (3, 71) = 7.60, *P* < 0.001, partial eta-squared = 0.243; and *F* (3, 71) = 18.32, *P* < 0.001, partial eta-squared = 0.436.

**Table 2:** Learning outcome comparison for offline and online groups

	Offline		Online		
Type of items	LD (*n* = 12)	SSD (*n* = 20)	LD (*n* = 21)	SSD (*n* = 22)	*P*
Total score (25 points)	12.17 (2.98)	17.35 (2.85)	10.19 (4.19)	16.55 (2.99)	< .001
Verbatim (10 points)	6.92 (1.62)	9.25 (0.85)	7.05 (2.67)	8.50 (1.23)	< .001
Transfer (15 points)	5.25 (2.05)	8.10 (2.47)	3.14 (2.74)	8.05 (2.63)	< .001

Data are shown as mean values (standard deviations)

*LD* Lecture and Discussion, *SSD* Self-study and Discussion

For each group, total, verbatim type, and transfer type item scores are given

A post hoc analysis using the Bonferroni test was then conducted to compare the four groups. The SSD offline group (*M* = 17.35, *SD* = 2.85) had the highest total mean values, followed by the SSD online group (*M* = 16.55, *SD* = 2.99), the LD offline group (*M* = 12.17, *SD* = 2.98), and the LD online group (*M* = 10.19, *SD* = 4.19), *P*s < 0.01. However, there were no significant differences between the SSD offline and online groups, and there were also no significant differences between the LD offline and online groups.

The SSD offline group (*M* = 9.25, *SD* = 0.85) had the highest verbatim type item scores, *P*s < 0.05, followed by the SSD online group (*M* = 8.50, *SD* = 1.23), which had significantly higher scores than the LD online group (*M* = 7.05, *SD* = 2.67), *P* = 0.049; however, there was no significant difference between the LD online and offline groups (*M* = 6.92, *SD* = 1.62), *P* = 0.083, the SSD offline and online groups.

**Fig. 2:**
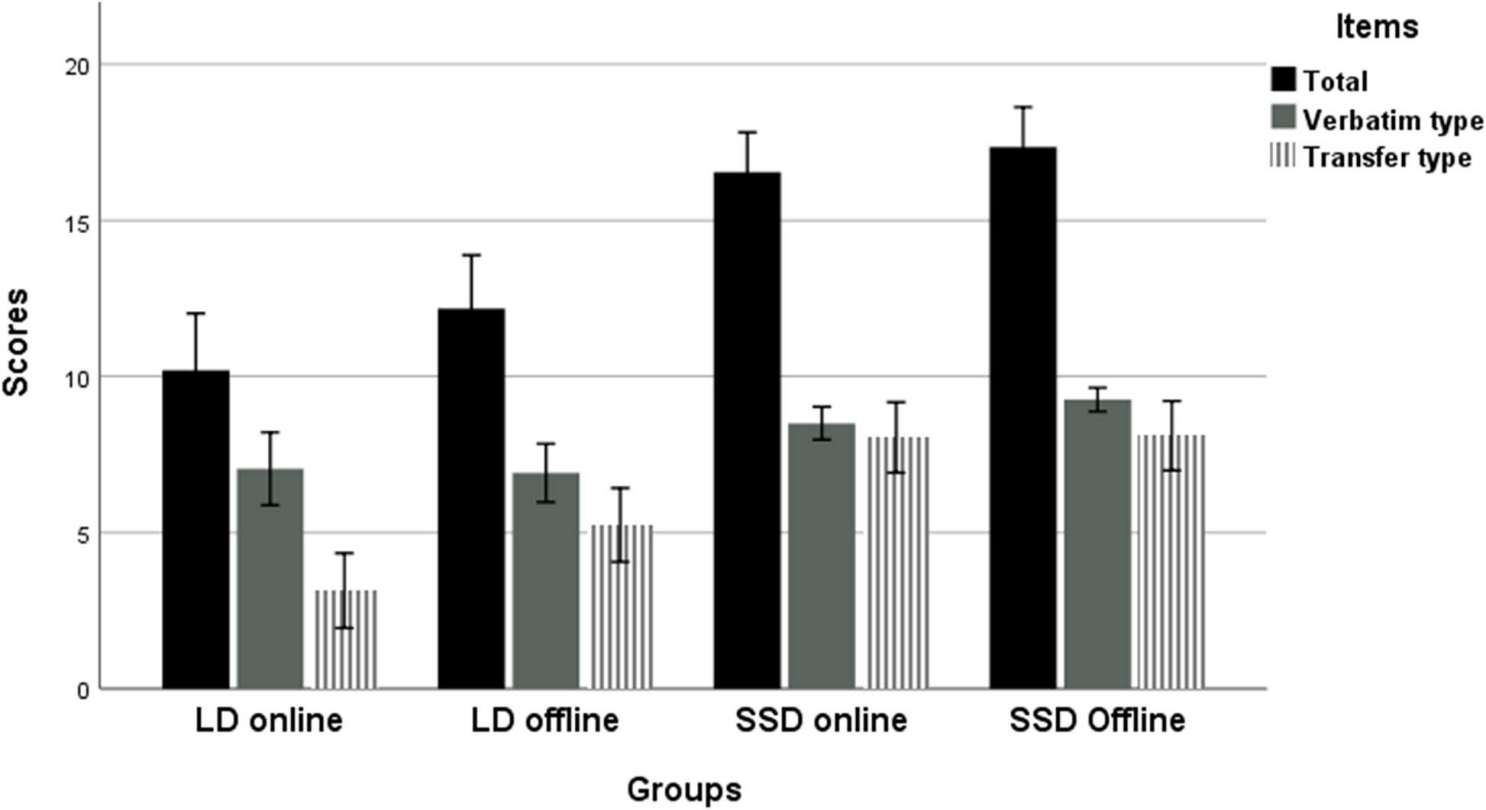
Summary of Offline and Online Studies. *Note*. LD = lecture and discussion group; SSD = self-study and discussion group. All types of items comprise both the verbatim and transfer type items. Error bars are ± 2standard errors

There was no significant difference between the transfer type item scores of the SSD offline (*M* = 8.10, *SD* = 2.47) and online (*M* = 8.05, *SD* = 2.63) groups; however, both were significantly higher than either the LD offline (*M* = 5.25, *SD* = 2.05) and the LD online (*M* = 3.14, *SD* = 2.74) groups, *P*s < 0.05. The LD online and the LD offline groups had no significant differences. Figure 2 shows a comparison of the offline study with the online study.

## Discussion

Although COVID-19 has focused attention on the need to care for patients and communities, there has been an insufficient focus on the COVID-19 effects on dental education. Thousands of schools and universities had to close to enforce social distancing, [4] which opened an opportunity to rethink education delivery systems.

To deal with the pandemic, many schools and higher educational institutions adopted online learning, such as web-based learning and e-learning. Although online learning has been a part of the education landscape for several decades, the developments in information technology have led to significant innovation opportunities. Accordingly, health professions education, such as dental education, began to investigate the feasibility of online education formats, and educators sought to minimize or eliminate one-way lectures and implement student-centered learning methods [7, 8]. Lim et al. proposed an active health professional learning method, which included dental education [8], confirmed the effectiveness of learning through discussions, and found that using active pre-discussion learning activities was the most effective way for students to learn. The experiment found that the more active the students were in the preliminary activities, the greater the effect of the discussion and the higher the student learning performances. Therefore, this study sought to gather additional evidence on the effectiveness of discussions with dentistry students by replicating the previous study in an online environment.

It was found that the students who had been involved in active learning activities  before the discussions had better outcomes than those who studied passively. While the main effect of the study condition (lecture versus. self-study) was significant, the main effect of the discussion condition (discussions with worksheet versus. free discussions) was not significant; however, the interaction effect of these two factors was found to be significant. Specifically, we found that the SSD group had the best performances for both the verbatim and transfer type items in the final test. There were no significant differences between the other three groups; however, the LW group had the lowest score. The SSD group had the highest verbatim type item scores, followed by the LD, SW, and LW groups, and for the transfer type items, the scores were also highest in the SSD group, followed by the SW group and the two lecture groups, which had no significant differences. These scores and patterns between the groups were similar for all types of items, which suggested that active learning activities enhance the effect of discussions and lead to higher learning outcomes. McNamara et al. suggested that before discussions, students need to be primed in a basic understanding of the learning content [37].

Structured discussions, including instructor intervention, such as a worksheet, had different effects depending on the learning activities before the discussion. For example, the LW group that used the worksheet had a lower score than the LD group that did not, and the SW group that used the worksheet had a lower score than the SSD group that did not. Of the two groups that listened to the lecture, the instructors’ intervention was found to have some positive effects on the student test scores; however, in the two self-study groups, the instructors’ intervention appeared to lower the student scores. These results suggested that free discussions, such as answering each other’s questions and discussing topics in more depth, are more effective for self-study groups, but that instructor intervention in lecture groups allows for deeper learning. These results support previous studies that found that one-way lectures did not lead to deeper learning than other methods [38–40].

The results for the dental students in the offline experiment were then compared to the online experiment. It was found that the test scores for the SSD offline group were higher than those for the SSD online group, both of which were much higher than the LD offline and online groups, which had no significant differences. The SSD offline group had the highest verbatim type item scores followed by the SSD online group, both of which were much higher than the LD offline and LD online groups, which had no significant differences. The SSD offline group had the highest transfer type item scores and the LD online group had the lowest. These comparison results indicated that regardless of whether the learning was offline or online, the groups that had discussions preceded by active learning (i.e., self-study, SSD group) had better learning performances than the LD groups, which confirmed that more active learning processes lead to a better understanding of the learning materials. Although the difference was not significant, the online groups generally scored lower than the offline groups. However, it was surmised that this could have been because of a lack of familiarity with the online platform and the active learning online process; therefore, it is expected that when students have more online learning experiences, the results will be similar to those of offline learning. What is key, however, is that there is adequate transfer of learning, the goal of education [41], and that this is more effective in self-study/discussion groups in both online and offline environments.

The results of the study in this study support Chi et al.’s ICAP framework, which emphasizes active learning [19, 20]; that is, student engagement promotes better learning outcomes as was evidenced by the higher scores for the self-study and discussion groups (i.e., the SSD, SW, SSD offline, and SSD online groups) compared with those of the lecture and discussion groups (i.e., the LD, LW, LD offline, and LD online groups). Based on the results of this study, online active learning that includes discussions is expected to be developed for health-related majors such as medicine and dentistry (e.g., online team-based learning and online project-based learning). However, future studies are needed to expand and generalize the findings.

One limitation of the present study was that the experimental group was limited to dental students. Therefore, future studies need to recruit students from other health professions, such as medicine and veterinary medicine, to conduct comparative studies. In addition, it is also possible to conduct follow-up experiments using specific topics including microbiology and immunology, which are required subjects for dentists. The second limitation was that the offline and current online experimental processes were not the same. The offline experiments used a question-generation condition [8], but a new variable was added in this study, i.e., instructor intervention with worksheets, during the discussion. In the analysis, although the groups containing the question-generation and instructor-intervention variables were removed, the experimental conditions may have been affected. Therefore, depending on the situation, caution is needed in future studies as the appearance of new experimental variables in the discussion could affect the experimental process. However, the instructor’s intervention, the new variable in the present experiment, was meaningful as it was found that the instructor intervention needed to be specifically modified depending on the learning conditions. Finally, in this study, students' prior knowledge was identified with a simple background knowledge survey, but it is not known whether the students' performance before the experiment was essentially equal. Therefore, future experiments can identify students' basic learning levels through quantitative methods such as pre-tests, and then measure the changed learning outcomes after the experiment.

This study explored effective online learning methods for health professions education. As dentistry and other health-related students need to absorb a great deal of knowledge, they require effective learning methods to better remember, apply, and use this knowledge in a range of situations [41, 42]. Therefore, this study presented a learning method that could improve learning outcomes, that is, focused self-study followed by discussion. More importantly, this study explored online active learning and assessed whether the discussion method could be applied online.

## Conclusion

Health professions education has been necessary to respond to the COVID-19 pandemic. This study examined whether online education could be suitable for health professions education contexts, especially dental education. The findings in this study supported the ICAP framework and provide practical implications for health professions education. It was found that rather than listening to lectures offline or online, the best learning mode was self-study followed by discussion. When students actively participate in learning activities before discussions, there is greater learning. In addition, the effect of the instructor's intervention in the middle of the discussion varied depending on the pre-learning activities of discussion. Therefore, new approaches, such as developing and applying existing methods online, could lead to more effective and diverse educational methods for health professionals.

## Data Availability

The datasets generated and analyzed during the current study are not publicly available due to restrictions from the Institutional Review Board but are available from the corresponding author upon reasonable request.

## Abbreviations

LD: Lecture and discussion
LW: Lecture and discussion with instructor’s worksheet
SSD: Self-study and discussion
SW: Self-study and discussion with the instructor’s worksheet

## References

[1] Clark D (2002). Psychological myths in e-learning. Med Teach.

[2] Jacobson MJ (2004). Cognitive visualisations and the design of learning technologies. Int J Learn Technol.

[3] Khan BH. Managing e-learning: Design, delivery, implementation, and evaluation. IGI Global. 2005.

[4] Toquero CM. Challenges and opportunities for higher education amid the COVID-19 pandemic: The Philippine context. Pedagogical Res. 2020;5(4). 10.29333/pr/7947.

[5] Fox R (2004). SARS epidemic: Teachers’ experiences using ICTs. in Beyond the comfort zone: Proceedings of the 21st ASCILITE Conference:319–327Citeseer.

[6] Koole S, Vervaeke S, Cosyn J, De Bruyn H (2014). Exploring the relation between online case- based discussions and learning outcomes in dental education. J Dent Educ.

[7] Rose S (2020). Medical student education in the time of COVID-19. JAMA.

[8] Lim J, Ko H, Yang JW (2019). Active learning through discussion: ICAP framework for education in health professions. BMC Med Educ.

[9] Irby DM, Cooke M, O’Brien BC (2010). Calls for reform of medical education by the Carnegie foundation for the advancement of teaching: 1910 and 2010. Acad Med.

[10] Kumar V, Gadbury-Amyot CC (2012). A case-based and team-based learning model in oral and maxillofacial radiology. J Dent Educ.

[11] Skochelak SE, Stack SJ (2017). Creating the medical schools of the future. Acad Med.

[12] Ihm J, Shin Y, Seo DG (2020). Did clinical reasoning and knowledge questions during team- based learning enhance dental students’ performance in esthetic dentistry?. J Dent Educ.

[13] Tucker B (2012). The flipped classroom. Educ Next.

[14] Takeuchi H, Omoto K, Okura K (2015). Effects of team-based learning on fixed prosthodontic education in a Japanese school of dentistry. J Dent Educ.

[15] Owen C, Ryall MA, Corrigan G (2007). Case-based learning: developing patient-and student- centred learning. Med Educ.

[16] Lum-Peng L, Ai-Yen C (1999). Challenges and relevance of problem-based learning in dental education. Eur J Dent Educ.

[17] Daradoumis T, Bassi R, Xhafa F, Caballé S. A review on massive e-learning (MOOC) design, delivery and assessment. In 2013 eighth international conference on P2P, parallel, grid, cloud and internet computing. 2013. p. 208-213.

[18] Hew KF, Cheung WS (2014). Students’ and instructors’ use of massive open online courses (MOOCs): motivations and challenges. Educ Res Rev.

[19] Chi MT, Wylie R (2014). The ICAP framework: linking cognitive engagement to active learning outcomes. Educ Psychol.

[20] Chi MT (2009). Active-constructive-interactive: a conceptual framework for differentiating learning activities. Top Cogn Sci.

[21] Murphy PK, Firetto CM, Wei L, Li M, Croninger RM (2016). What REALLY works: optimizing classroom discussions to promote comprehension and critical-analytic thinking. Policy Insights Behav Brain Sci.

[22] Murphy PK, Greene JA, Firetto CM (2018). Quality talk: developing students’ discourse to promote high-level comprehension. Am Educ Res J.

[23] Langer JA (2000). Excellence in English in middle and high school: how teachers’ professional lives support student achievement. Am Educ Res J.

[24] Langer JA (2001). Beating the odds: teaching middle and high school students to read and write well. Am Educ Res J.

[25] Mujayanto R, Indraswary R (2020). Differential diagnosis of COVID-19 enanthema. Eur J Dent.

[26] Meng L, Hua F, Bian Z (2020). Coronavirus disease 2019 (COVID-19): emerging and future challenges for dental and oral medicine. J Dent Res.

[27] Usak M, Masalimova AR, Cherdymova EI, Shaidullina AR (2020). New playmaker in science education: Covid-19. J Balt Sci Educ.

[28] Ruberg LF, Moore DM, Taylor CD (1996). Student participation, interaction, and regulation in a computer-mediated communication environment: a qualitative study. J Educ Comput Res.

[29] Warschauer M (1997). Computer-mediated collaborative learning: theory and practice. Mod Lang J.

[30] Corey SM (1934). Learning from lectures vs. learning from readings. J Educ Psychol.

[31] Lee J, Choi H (2019). Rethinking the flipped learning pre-class: Its influence on the success of flipped learning and related factors. Br J Edu Technol.

[32] Faul F, Erdfelder E, Lang AG, Buchner A (2007). G* Power 3: a flexible statistical power analysis program for the social, behavioral, and biomedical sciences. Behav Res Methods.

[33] Beyer BK. Practical strategies for the teaching of thinking. Newton: Allyn and Bacon; 1987.

[34] Miyake N, Norman DA (1979). To ask a question, one must know enough to know what is not known. J Verbal Learn Verbal Behav.

[35] Barnett SM, Ceci SJ (2002). When and where do we apply what we learn?: a taxonomy for far transfer. Psychol Bull.

[36] Cook DA, Levinson AJ, Garside S, Dupras DM, Erwin PJ, Montori VM (2008). Internet-based learning in the health professions: a meta-analysis. JAMA.

[37] McNamara DS, Kintsch E, Songer NB, Kintsch W (1996). Are good texts always better? inter-actions of text coherence, background knowledge, and levels of understanding in learning from text. Cogn Instr.

[38] Hrepic Z, Zollman DA, Rebello NS (2007). Comparing students’ and experts’ understanding of the content of a lecture. J Sci Educ Technol.

[39] Wieman C, Perkins K (2005). Transforming physics education. Phys Today.

[40] Poh MZ, Swenson NC, Picard RW (2010). A wearable sensor for unobtrusive, long-term assessment of electrodermal activity. IEEE Trans Biomed Eng.

[41] Goldstone RL, Day SB (2012). Introduction to “new conceptualizations of transfer of learning”. Educ Psychol.

[42] Moon SH, Myung SJ, Yoon HB, Park JB, Kim JW, Park WB (2019). Deliberate practice as an effective remediation strategy for underperforming medical students focused on clinical skills: a prospective longitudinal study. J Korean Med Sci.

